# Development and validation of a realistic type III esophageal atresia simulator for the training of pediatric surgeons

**DOI:** 10.1007/s00383-024-05827-5

**Published:** 2024-09-09

**Authors:** Javier Arredondo Montero, Blanca Paola Pérez Riveros, Oscar Emilio Bueso Asfura, Nerea Martín-Calvo, Francisco Javier Pueyo, Nicolás López de Aguileta Castaño

**Affiliations:** 1 Pediatric Surgery Department, Complejo Asistencial Universitario de León, c/Altos de Nava s/n, 24008 León, Castilla y León, Spain; 2 Department of Preventive Medicine and Public Health, School of Medicine, University of Navarra, c/Irunlarrea 1, 31008 Pamplona, Navarra, Spain; 3 IdiSNA, Instituto de Investigación Sanitaria de Navarra, Pamplona, Navarra, Spain; 4 CIBER de Fisiopatología de la Obesidad y la Nutrición, Instituto de Salud Carlos III, Madrid, Spain; 5 Department of Anesthesiology, University of Navarra Clinic Pamplona, Navarra, Spain; 6 Medical Engineering Laboratory, School of Medicine, University of Navarra, Pamplona, Navarra, Spain

**Keywords:** Esophageal atresia, Simulation, Silicone, 3D model, Pediatric surgery, Training, Model

## Abstract

**Background:**

The technical complexity and limited casuistry of neonatal surgical pathology limit the possibilities of developing the necessary technical competencies by specialists in training. Esophageal atresia constitutes the paradigm of this problem. The use of synthetic 3D models for training is a promising line of research, although the literature is limited.

**Methods:**

We conceptualized, designed, and produced an anatomically realistic model for the open correction of type III oesophageal atresia. We validated it with two groups of participants (experts and non-experts) through face, construct, and content-validity questionnaires.

**Results:**

The model was validated by nine experts and nine non-experts. The mean procedure time for the experts and non-experts groups was 34.0 and 38.4 min, respectively. Two non-experts did not complete the procedure at the designed time (45 min). Regarding the face validity questionnaire, the mean rating of the model was 3.2 out of 4. Regarding the construct validity, we found statistically significant differences between groups for the equidistance between sutures, 100% correct in the expert group vs. 42.9% correct in the non-expert group (*p* = 0.02), and for the item “Confirms that tracheoesophageal fistula closure is watertight before continuing the procedure”, correctly assessed by 66.7% of the experts vs. by 11.1% of non-experts (*p* = 0.05). Concerning content validity, the mean score was 3.3 out of 4 for the experts and 3.4 out of 4 for the non-experts.

**Conclusions:**

The present model is a cost-effective, simple-to-produce, and validated option for training open correction of type III esophageal atresia. However, future studies with larger sample sizes and blinded validators are needed before drawing definitive conclusions.

**Supplementary Information:**

The online version contains supplementary material available at 10.1007/s00383-024-05827-5.

## Introduction

Surgery is a constantly developing field. The advent of minimally invasive techniques and highly complex procedures (such as robotic surgery or microsurgery) has significantly improved patients’ overall health outcomes and perioperative morbidity. Still, it has also considerably increased the learning curves of the procedures [1–4]. In pursuit of the best patient outcome, the global trend has been to centralize these procedures in high-volume centers [5, 6]. While this has proven beneficial in multiple pathologies, it has also prevented many of these procedures from being trained and learned by the bulk of surgical specialists. This is especially relevant in Pediatric  Surgery, where we find additional challenges such as the reduced size of the surgical fields, the tissue fragility, or the intrinsic lability of young patients regarding homeostasis, which requires procedures to be carried out quickly and efficiently. And all these challenges reach their highest expression in neonatal surgery.

One of the main problems in neonatal surgery is the limited caseload. Although it varies according to pathology, finding fewer cases than estimated necessary to acquire basic skills in managing these pathologies is common. The absence of standardized guides for trainees specifying how many procedures of each pathology they must attend and perform before the end of their training period also contributes to this problem. In addition, the development of minimally invasive techniques in neonatal surgery (such as thoracoscopic correction of esophageal atresia) has led to a tendency for these pathologies to be centralized and operated by senior consultants, limiting the training possibilities for residents. Finally, the existence of different residency programmes depending on the country also conditions the acquisition of these competences. There are countries where pediatric surgery is a complete surgical specialty with a 5-year training (like Spain), while in other countries, Pediatric Surgery is a fellowship derived from general surgery [7, 8].

Of all neonatal surgical pathologies, esophageal atresia constitutes the paradigm: its high technical complexity, the potential sequelae of surgical iatrogenesis, and the need for a fast and efficient procedure due to the respiratory and hemodynamic lability of the patients justify the need for prior targeted training before performing the procedure in humans. There are few precedents for neonatal thoracic and esophageal atresia models for surgical training [9–15]. Under this premise, we conceptualized, developed, and validated the present model of type III esophageal atresia (esophageal atresia with distal tracheoesophageal fistula).

## Methods

### Conceptualization and preliminary design of the model

**Fig. 1:**
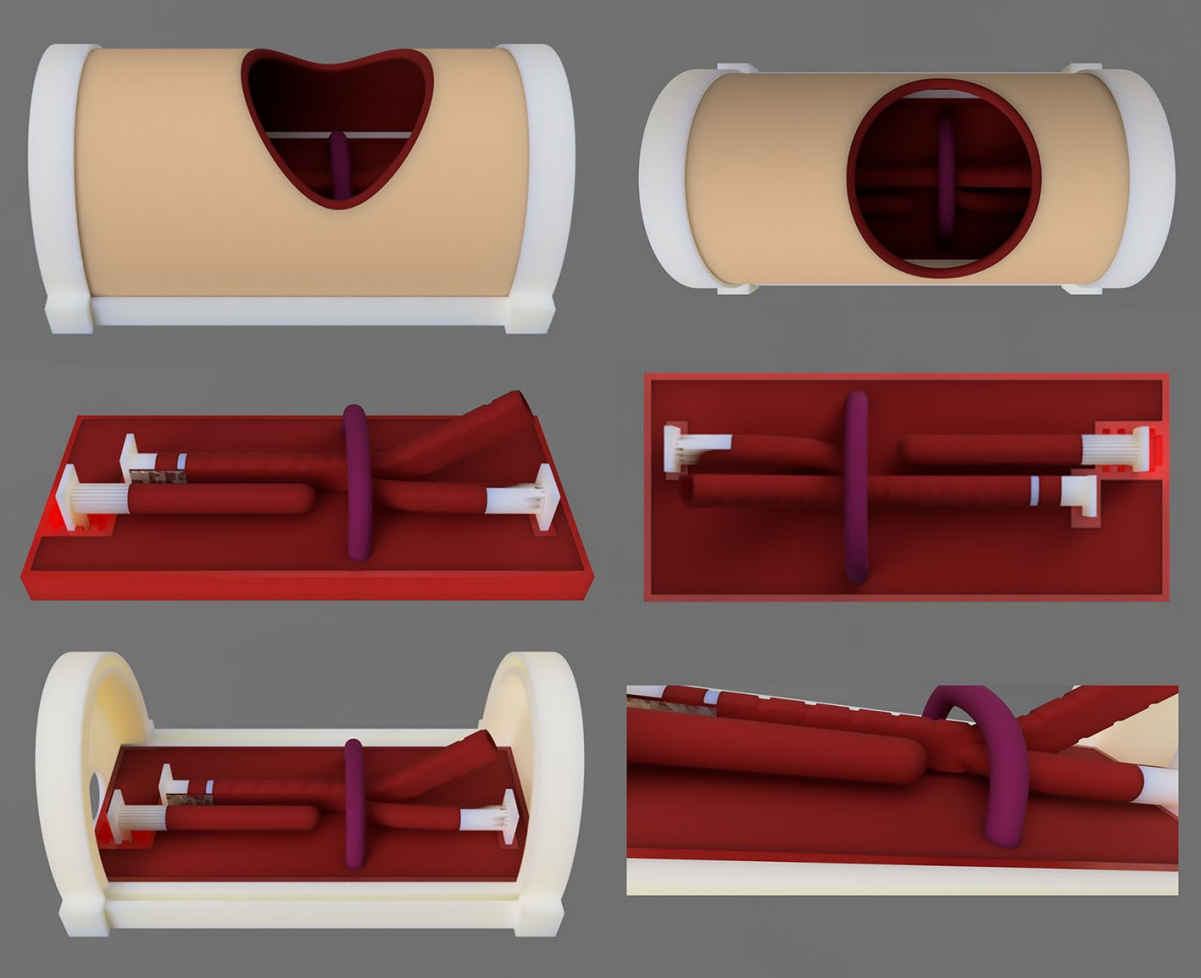
Images corresponding to the model design phase, made in Autodesk Fusion 360^®^ (Autodesk, CA., USA)

For the initial design, anthropometric references of the esophagus, the azygos vein, and the trachea were taken from different bibliographic references [16, 17], and a preliminary range of measures and diameters was established. Autodesk Fusion 360^®^ (Autodesk, CA., USA) was used to design the model’s first topographic composition and establish an approximate proportional relationship between the different elements that integrated it. This modeling phase was characterized by iterative adjustments and refinements, ensuring the model’s high fidelity to neonatal esophageal conditions (Fig. 1).

### Model production methodology

The simulator's soft base was created with 50 g of Eco-Flex 0030 silicone dyed red. 3D-printed components, including the bases for the esophagus, trachea, and side caps, were produced using polylactic acid (PLA) (Smart Materials 3D, Jaén, Spain) using a Prusa MK3S 3D printer (Prusa Research, Prague, Czech Republic). 3D-printed materials were post-processed as required.

The trachea was molded with a Dragon Skin 10 medium silicone (1A:1B, 20 g total) with red dye, and the esophagus was molded with an Eco-Flex 0030 platinum silicone with red dye (1A:1B, 5 g total). A silicone-based model was chosen instead of a rigid 3D impression to allow a realistic flexible fibro bronchoscopy before the procedure (Supplementary Material 1) and a realistic closure of the tracheo-oesophageal fistula.

The torso was molded using a Sorta Clear bicomponent silicone with red dye (1A:1B, 150 g total), covered by a layer of Eco-Flex 0030 platinum silicone with ‘flesh’ pigment and Eco-Flex 35 Fast silicone for a realistic skin texture. The azygos vein was molded using the same silicone mixture as the esophagus but with different mixtures (1A:1B, 10 g total).

During the assembly process, the silicone torso was attached to the side covers and trimmed to fit correctly. The tracheoesophageal fistula was manually made, ensuring a homogeneous distribution of the silicone sealant. The trachea and the esophagus were inserted into the main base and secured with nylon flanges. The azygos vein was positioned in the desired position by drilling into the silicone and PLA bases and inserting the silicone tube into the holes. Finally, it was filled with water, tinted with a drop of blue methylene, and sealed.

### The final version of the model

The present model is constituted by a rectangular surface padded with silicone on which is installed a plastic frame that anchors the essential elements of the model: (1) an anatomically realistic, neonatal-sized trachea, with tracheal rings, a carina, and two main bronchi with a well-delimited *Pars Membranosa* (of 0.8 mm). The internal diameter of the trachea was 8 mm. The external diameter was 10.4 mm at the level of the tracheal rings and 9.6 mm at the level of the space between tracheal rings, and (2) an atretic esophagus with a blind proximal pouch and a distal tracheoesophageal fistula inserted into the trachea with a permeable lumen and conical morphology, simulating actual surgical conditions. The esophagus has an outer diameter of 8 mm and an inner diameter of 6 mm. (3) An azygos vein located over the distal tracheoesophageal fistula, with an internal diameter of 5 mm, filled with water with dye (methylene blue). 

**Fig. 2:**
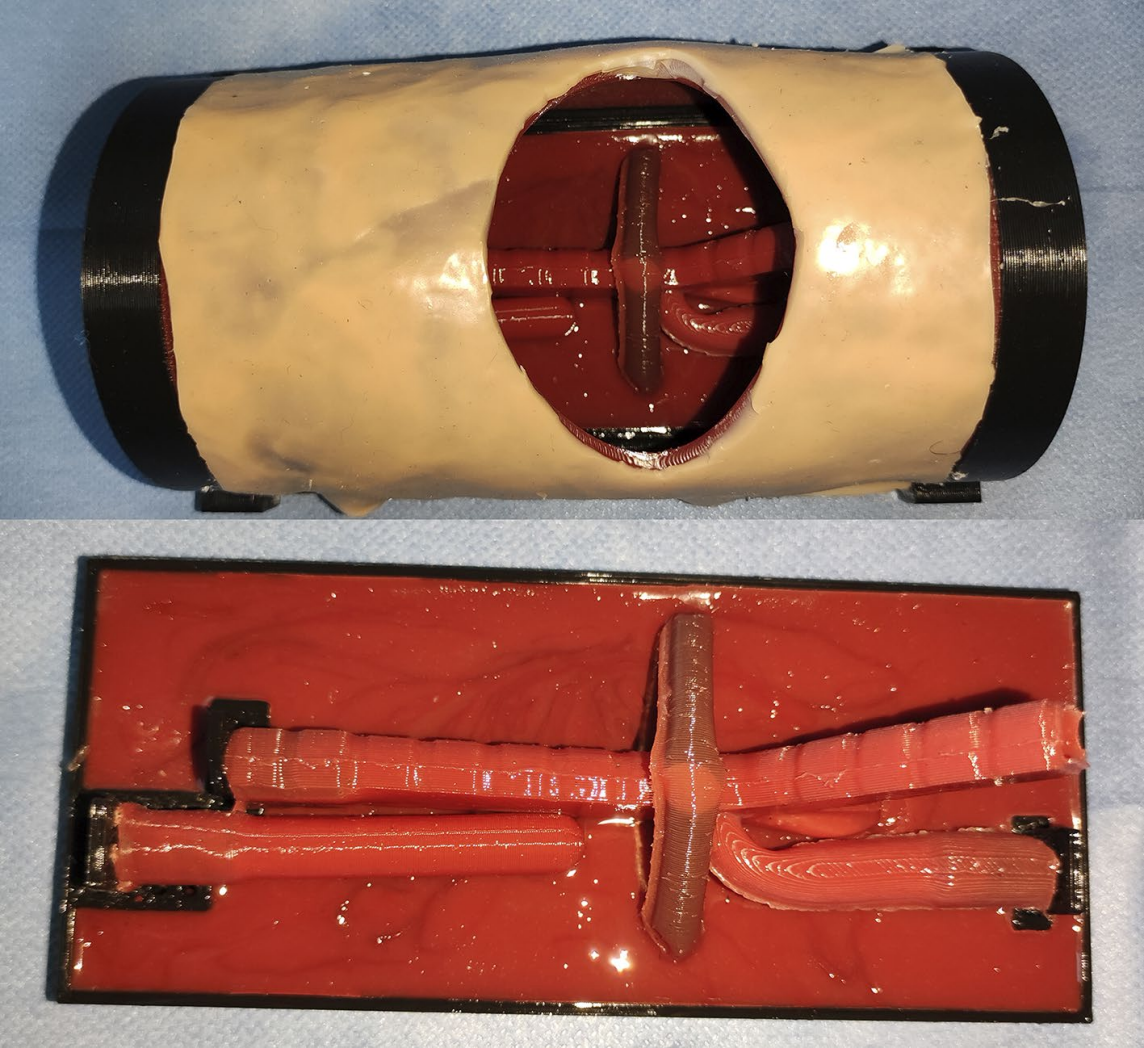
Above: the final model appearance with thoracic coverage. Below: the final model appearance without thoracic coverage

**Fig. 3:**
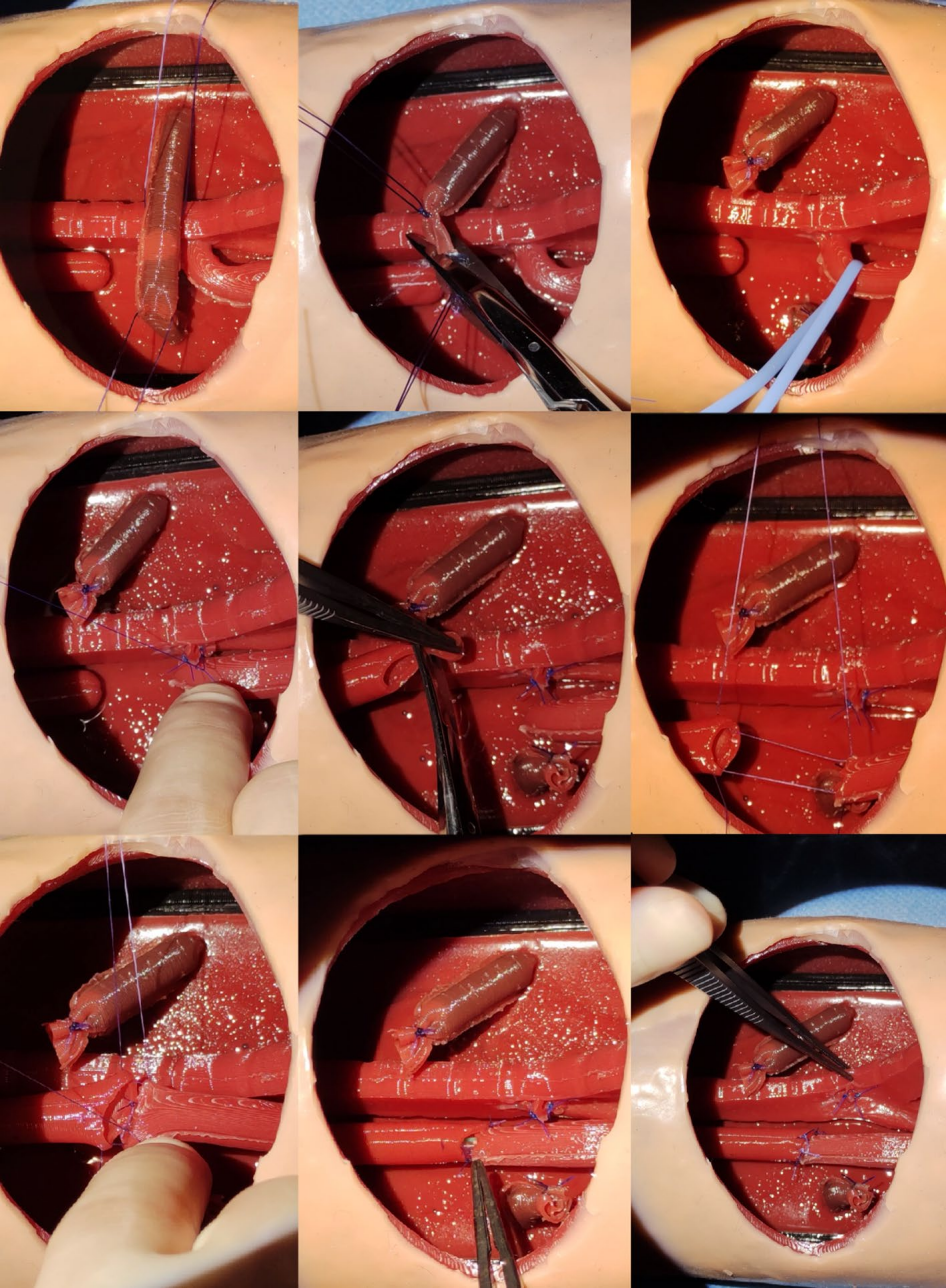
Representative images of the surgical procedure performed on the model. Above left, center: azygos vein reference and ligation; above right, center left: tracheoesophageal fistula reference and ligation. Center, center: opening of the upper esophageal pouch; center, right: lateral sutures of the oesophageal anastomosis; bottom left, center: oesophageal anastomosis. Bottom, right: final result

The model allows regulation of the separation between the two esophageal ends to perform the procedure with different degrees of tension and allows a flexible bronchoscopy with a fibrobronchoscope up to 4 mm (Supplementary File 1). Externally, the model is covered by a convex multilayer semi-rigid silicone that simulates the neonatal thorax. On this silicone, performing any desired approach (either thoracoscopic or using a variable-size thoracotomy) is possible. The simulator's dimensions are 146 mm × 82 mm × 63 mm (Figs. 2, 3).

The production time for the complete model is 3 h, and for the replacement parts (silicone parts), it is 2 h. The production cost of the model, considering materials, labor, and indirect costs (electricity consumption), is 52.58 € for the complete model and 35.86 € for the model without the molds. The cost of each spare part (azygos vein, esophagus, and trachea) is 17.05 €.

### Validation

A validation protocol was designed and implemented with two groups of validators: (1) experts (group 1) and (2) non-experts (group 2). Experts consisted of consultant Pediatric Surgeons (PS) who had performed the procedure at least once. The non-expert group was required to have basic surgical skills, but no specific training in neonatal surgical pathology or in the surgical management of oesophageal atresia. Therefore, the non-expert group consisted of second-to-fifth year General or Pediatric Surgery residents.

The validators (OEB and BPR) were specifically instructed in the pathology, surgical technique, validation questionnaires, and elements to be assessed during the procedure. They supervised all validations.

Both experts and non-experts had never encountered the model before. Specific questionnaires and checklists were designed for construct, content, and face validity in the validation process.

The validation process had the following structure: (1) first, an illustrative video was reproduced, showing with detail the surgical procedure to be performed with a demonstration of the use of the model (Supplementary File 2); (2) validation was initiated under direct visual supervision of the validators and with continuous recording of the surgical field in case a re-evaluation of the validations was required.

## Results

Nine experts (group 1) and nine non-experts (group 2) validated the model. The mean procedure time for the experts and non-experts groups was 34 (sd = 5.8) and 38.4 (sd = 6) min, respectively. Two non-experts did not complete the procedure within the proposed time limit, which was 45 min.

### Construct validity

All the experts (*n* = 9) and non-experts (*n* = 9) responded to the construct validity questionnaire. We found statistically significant differences for the equidistance between sutures, which was 100% correct in the expert group vs. 42.9% correct in the non-expert group (*p* = 0.02) and for the item “Confirms that tracheoesophageal fistula closure is watertight before continuing the procedure”, which was correctly assessed by 66.7% of the experts vs. by 11.1% of non-experts (*p* = 0.05). Table 1 shows the comparison between groups for construct validity items.

**Table 1:** Construct validity evaluation

Items	Experts (*n* = 9)	Non-experts (*n* = 9)	*p*-value
Decides to ligate the azygos vein before dissecting the esophagus (% yes)	100	100	
Ligates the azygos vein without tearing it (% yes)	77.8	77.8	0.99
Correctly identifies both esophageal pouches and tracheoesophageal fistula (% Yes)	88.9	88.9	0.99
Checks by extrinsic collapse that the tracheoesophageal fistula has been correctly identified (% Yes)	66.7	22.2	0.15
Closes tracheoesophageal fistula progressively (cut-suture-cut) (% Yes)	77.8	88.9	0.99
Resects only the essential amount of esophagus at the level of the tracheoesophageal fistula (% Yes)	100	88.9	0.99
Confirms that tracheoesophageal fistula closure is watertight before continuing the procedure (% Yes)	66.7	11.1	0.05
Ensures that there are no additional tracheoesophageal fistulas (% Yes)	77.8	55.6	0.62
Adequately dissects both esophageal ends (% Yes)	100	100	
Checks the distance between esophageal ends and the viability of the anastomosis before performing the anastomosis (% Yes)	88.9	55.6	0.29
Starts with side sutures and references them (% Yes)	100	100	
Then performs the posterior layer of the anastomosis (% Yes)	100	88.9	0.99
Places a trans-anastomotic tube before suturing the anterior layer (% Yes)	77.8	55.6	0.62
In general, lowers the knots with a controlled and homogeneous tension and accompanies them by the dominant finger (% Yes)	100	88.9	0.99
In general, sutures are total thickness (all layers) (% Yes)	100	87.5	0.47
In general, sutures are equidistant from each other (% Yes)	100	42.9	0.02
In general, adequately lowers knots (“surgeon´s knots”) (% Yes)	100	100	
Completes the procedure (% Yes)	100	77	0.47
Mean number of sutures used	3.1	3.7	0.35
Mean procedure time (minutes)	34	38.4	0.19

### Face validity

All the experts (*n* = 9) responded to the face validity questionnaire. All items were rated on a scale from 1 (worst) to 4 (best). The best-rated aspect was “this model allows the acquisition of surgical skills transferable to the real surgical field”, with a score of 3.7. The worst-rated item was “the azygos vein resembles that of a neonate”, with a score of 2.4. The mean rating of the model was 3.2 out of 4 (sd = 0.27). Table 2 shows the mean score attributed to each item in the questionnaire.

**Table 2:** Face validity questionnaire

Item	Mean score (experts) *N* = 9
The esophageal diameter of the model resembles that of type III esophageal atresia (esophageal atresia with distal tracheoesophageal fistula)	3.4
The esophageal thickness of the model resembles that of type III esophageal atresia (esophageal atresia with distal tracheoesophageal fistula)	3.3
The trachea resembles that of a neonate	2.9
The azygos vein resembles that of a neonate	2.4
The elements of the model are adequately represented and laid out	3.1
The layers of the model realistically simulate an actual esophagus	3.2
Visually, the model material resembles an actual esophagus	3
The model's feel (wet and slippery) is reminiscent of an esophagus	3.1
The texture and consistency of the model when the suture needle passes through it are similar to that of an esophagus	3.3
The sensation of suturing the ends of the model is similar to that of an esophagus	3.1
The model reproduces the surgical dimensions of a neonatal thoracic field	3.4
The spatial positioning of the model resembles that of a surgical field	3.1
The model allows the mobility of the structures to be similar to reality	3
In general, the execution of the surgical technique for the correction of type III esophageal atresia on the model resembles reality	3.3
This model is useful for learning the surgical technique for correcting type III esophageal atresia	3.4
This model allows a realistic simulation of an end-to-end esophageal anastomosis (moderate tension)	3.3
This model allows the acquisition of surgical skills transferable to the real surgical field	3.7
This model realistically reproduces the level of difficulty of the procedure	3.1
Mean score	3.2

Each item was evaluated on a 4-point scale (best rating 4, worst rating 1)

### Content validity

All the experts (*n* = 9) and non-experts (*n* = 9) responded to the content-validity questionnaire. All items were rated on a scale from 1 (worst) to 4 (best). In the expert’s group, the best-rated aspect was “this model helps the user understand the technique behind the procedure"”, with a mean score of 3.8, and the worst-rated aspect was “this model measures the user's ability to perform corrective surgery for esophageal atresia type III (esophageal atresia with distal tracheoesophageal fistula)” with a mean score of 2.9. In the non-experts group, the best-rated aspect was “this model allows to learn different surgical techniques,” with a mean score of 3.8, and the worst-rated aspect was “this model measures the user's ability to perform corrective surgery for esophageal atresia type III (esophageal atresia with distal tracheoesophageal fistula)” with a mean score of 3. The mean rating of the model was 3.3 out of 4 for the experts (sd = 0.28) and 3.4 out of 4 for the non-experts (sd = 0.22). Table 3 shows the mean score attributed to each item in the questionnaire.

**Table 3:** Content validity questionnaire

Item	Mean score (experts) *N* = 9	Mean score (non-experts) *N* = 9	*p*-value
This model helps the user understand the technique behind the procedure	3.8	3.4	0.54
This model helps the user understand the spatial arrangement of the thorax and mediastinum in a neonate	3.4	3.3	0.64
This model helps the user learn how to handle the esophagus and neonatal mediastinal structures in a surgical context	3	3.2	0.33
This model helps the user understand how esophageal and tracheal tissue respond to being handled in surgery	3	3.3	0.12
This model helps the user understand how esophageal tissue responds to an end-to-end anastomosis under moderate tension	3.2	3.3	0.76
This model allows to learn different surgical techniques	3.2	3.8	0.11
This model allows the training of different surgical techniques	3.6	3.6	0.78
This model allows to evaluate the user's surgical technique	3.2	3.3	0.76
This model measures the user's ability to perform corrective surgery for esophageal atresia type III (esophageal atresia with distal tracheoesophageal fistula)	2.9	3	0.65
This model helps the user to be better prepared when performing corrective surgery for esophageal atresia type III (esophageal atresia with distal tracheoesophageal fistula) in a neonate for the first time	3.6	3.6	0.8
This model increases the user's confidence before performing corrective surgery for type III esophageal atresia (esophageal atresia with distal tracheoesophageal fistula) in a neonate	3.4	3.6	0.65
Mean score	3.3	3.4	

Each item was evaluated on a 4-point scale (best rating 4, worst rating 1)

## Discussion

Through the present work, our pediatric surgical simulation development group (SIMUPED^®^) conceptualized, designed, produced, and validated an anatomically realistic model of type III oesophageal atresia for the training of Pediatric Surgeons. The limited casuistry of this pathology, the lability of the patients who suffer from it, and the high technical complexity of the corrective procedure justify the development of training models to acquire the necessary skills and competencies before performing the procedure on humans. The model we have developed stands out for being anatomically realistic, having a low production cost (both in terms of time and materials), and allowing flexible bronchoscopy to be performed.

In this validation study, no differences were found in most items between experts and non-experts for the construct validity items, except for the equidistance between sutures and the confirmation of the watertight closure of the tracheoesophageal fistula. We attribute these findings to the fact that this procedure is highly technical and specialized and requires extensive pre-procedural guidance and instructions. This pre-procedural guidance may have hampered our results. Notably, two non-experts did not complete the procedure in time, which also may have hampered our results. If we considered a hypothetical completion time of 46 min for these two non-experts (one minute more than the proposed limit), the difference in time between the two groups would have reached marginal significance (*p* = 0.06). The face validity obtained an overall positive evaluation, scoring 3.2 out of 4. The lowest-rated item, the azygos vein anatomy, corresponds to the most challenging structure to replicate due to the technical limitation of generating an anatomically realistic hollow tubular structure filled with liquid. However, we consider that its consistency and the presence of dyed liquid inside it allows to train the dissection and ligation technique effectively. Finally, concerning content validity, the overall assessment of the model was positive, with an average of 3.3 out of 4 in the group of experts and 3.4 out of 4 in the group of non-experts. The fact that the most highly rated item by the experts was that the model allowed an understanding of the technique is very positive in terms of the transferability of the model.

The validation study faced some difficulties worth mentioning. (1) Firstly, establishing the definition of experts and non-experts was complicated: the non-experts must have enough basic surgical skills to be able to carry out the procedure, but they had to be sufficiently different from the experts so that the model could distinguish them in the construct validity questionnaire. It may be that in our case the groups were not sufficiently different. In addition, the questions included in the construct validity questionnaire may not have accurately reflected the expert character. However, we found significant differences in items of great relevance (such as the equidistance between sutures), which supports the constructive validity of the model. (2) Secondly, due to the scarce number of experts available in our area, the recruitment of participants for this group was significantly limited.

To our knowledge, seven models of oesophageal atresia have been previously published, all of them between 2014 and 2023. A summary of the main characteristics of the models published to date is shown in Table 4. Except for the first one, developed by Barsness et al. and partially made of bovine fetal tissue, all subsequent ones have been synthetic. Regarding the surgical technique aimed, the model by Neville et al. was designed expressly for open surgery, while all the others were intended for thoracoscopic procedures. To our knowledge, the one presented in this work is the first model that allows the performance of a realistic flexible bronchoscopy. This involves multiple aspects of interest, such as specific training in neonatal bronchoscopy or endoscopic treatment of tracheoesophageal fistula. Variants of this model aimed at training and developing these competencies are of interest in future. One of our model’s strengths is using silicone to replicate the trachea because it results in a more realistic consistency than 3D-printed ones. Another highlight of our model is that since the tracheoesophageal fistula is made of silicone and placed manually, all subtypes of esophageal atresia could be simulated by introducing slight variations in the model. This is of great value if the trainee wants to perform the procedure blind to the subtype of esophageal atresia, which is where most surgeons find themselves before starting an operation of this type. In addition to having a lower cost, another essential contribution of our model compared to the existing ones is the inclusion of the azygos vein. The possibility of regulating the distance and tension of the oesophageal ends is a notable contribution to the previous models since it allows for the variation of the difficulty of the practice. In our experience, we have performed high-tension anastomosis with the model (simulating a long-gap esophageal atresia) and the procedure is technically feasible although it presents much greater technical difficulty.

**Table 4:** Summary of the esophageal atresia training models published to date

Author	Country	Model type	Technique	Validators	Validation items	Validation results	Model cost	Model measures
Barsness et al. [9]	USA	Mixed (synthetic + bovine fetal tissue)	Thoracoscopy	Non-experts: self-reported “novice” PS (*n* = 12) Experts: self-reported “experienced” PS (*n* = 8)	24 item content validity survey OSATS—two independent PS	High overall simulator ratings OSATS: high interitem consistency OSATS: high interrater agreement	NR	NR
Harada et al. [10]*	Japan	Synthetic	Thoracoscopy	PS (*n* = 4)	Optical and electromagnetic tracking Task completion time Content validity survey	High overall simulator ratings High tracking ratio (99.8%)	NR	NR
Maricic et al. [11]	Argentina	Synthetic	Thoracoscopy	Experts (PS with > 30 TEF/EA repairs) (*n* = 7) Intermediates (PS with 5 to 29 TEF/EA repairs) (*n* = 10) Beginners (PS with < 5 TEF/EA repairs) (*n* = 22) Total: *n* = 39	Likert-type scale Performance checklist	High overall simulator ratings Significant differences in time and number of errors between Experts/intermediates and beginners	Low cost (not specified)	NR
Deie et al. [12]*	Japan	Synthetic	Thoracoscopy	Experts (PS with ≥ 3 TEF/EA thoracoscopic repairs) (*n* = 6) Non-experts (PS with < 3 TEF/EA thoracoscopic repairs) (*n* = 34) Validators divided also between ESSQ qualified (*n* = 15) vs. ESSQ non-qualified (*n* = 25)	Questionnaire (from Barsness et al.) Video-based endoscopic surgical skill assessment: 29-item checklist Error assessment sheet	Experts significantly superior to non-experts	NR	NR
Neville et al. [14]	UK	Synthetic	Open repair	Experts (*n* = 12) Non-experts (*n* = 28)	Construct validity Content validity Face validity	High overall simulator ratings Experts significantly superior to non-experts	Structure: 100￡ Replaceable parts: 20￡ per use	NR
Zahradniková et al. [15]	Slovakia	Synthetic	Thoracoscopy	Experts (experienced surgeons): *n* = 7 Intermediates: pediatric surgery trainees (*n* = 4) Novices: medical students (*n* = 7)	Face validity Content validity Overall impression OSATS Quality of the anastomosis	High overall simulator ratings	NR	NR

*UK* United Kingdom; *USA* United States of America; *PS* pediatric surgeons; *OSATS* objective structured assessments for technical skills; *NR* not reported; *TEF/EA* esophageal atresia with tracheoesophageal fistula; *ESSQ* endoscopic surgical skill qualification; *ID* inner diameter; *OD* outer diameter

*Same working group

Our model is oriented to the open correction of atresia. Although the model allows the procedure to be performed thoracoscopically, we believe that it is essential that all Pediatric Surgeons receive adequate training in the open correction of oesophageal atresia. To the best of our knowledge, this is (together with that of Neville et al.) one of the first models oriented along these lines.

Lastly, although we considered the possibility of testing the anastomosis for leakage after the procedure was completed, the preliminary laboratory tests we performed showed frequent leakage through the needle insertion and passage points, and therefore, we considered that the leakage test was not feasible. We attributed this to the intrinsic characteristics of the silicone. This should be considered a limitation of our model and a potential area for improvement in subsequent designs.

Regarding the cost of our model, it should be considered that the price was estimated for individual hand-made production. The production process of our model is industrialisable, which would result in greater uniformity and lower labor costs. The price would become much more competitive since the bulk of the price we report here is labor.

We found significant heterogeneity in the validation studies of the previously published models. Although the reported results are generally good, we identified several discrepancies in the definition of the groups of experts and non-experts, the sample sizes, and the validation methodology used. Including a group of non-experts should be considered a strength of our work. We also believe that the design of construct validity questions is complex and would benefit in future from collaborative groups for their conception (e.g., through the Delphi method).

This study is not exempt from limitations. First, the limited sample size may have hampered the obtention of more statistically significant differences between groups. Second, the validators were not blinded to the type of participant, which may have biased their responses. Third, additional external validation would have been helpful to assess the reproducibility of the construct validity questionnaire. Finally, the definition of the expert and non-expert groups we proposed is debatable since considering a Pediatric Surgeon who has only performed the procedure once as an expert is probably insufficient. However, given the limitations of our environment, setting a higher cut-off point for experts would have made it more difficult to recruit them. In our opinion, the best group of non-experts would probably be defined as Pediatric Surgeons who have never performed this procedure since, in this case, they have the basic pediatric surgical skills and finesse but not the specific competencies for this particular procedure.

On the other hand, this study’s main strength is the detailed reporting of model design, production and validation, which facilitates its reproducibility.

In conclusion, the present model is a low-cost, simple-to-produce, and validated option for the training of open correction of type III oesophageal atresia. Future studies with larger sample sizes and blinded validators are needed to draw definitive conclusions.

## Supplementary Information

Below is the link to the electronic supplementary material.

Supplementary file 1: Flexible bronchoscopic examination performed on the present model. Left: Visualization of the carina; Right: Right: visualization of the carina with the tracheoesophageal fistula on the upper right side of the image (TIF 5063 KB)

Supplementary file 2: Video demonstration of the open surgical correction of esophageal atresia in the present model (Procedure carried out and filmed by JAM)  (MP4 1607051 KB)

## Data Availability

The data used to carry out this study are available upon request from the authors.
