# Supplementary figures and images for: Development and validation of a realistic type III esophageal atresia simulator for the training of pediatric surgeons
Source: Pediatr Surg Int. 2024 Sep 9;40(1):251. doi: 10.1007/s00383-024-05827-5 (PMC11385018; doi:10.1007/s00383-024-05827-5)

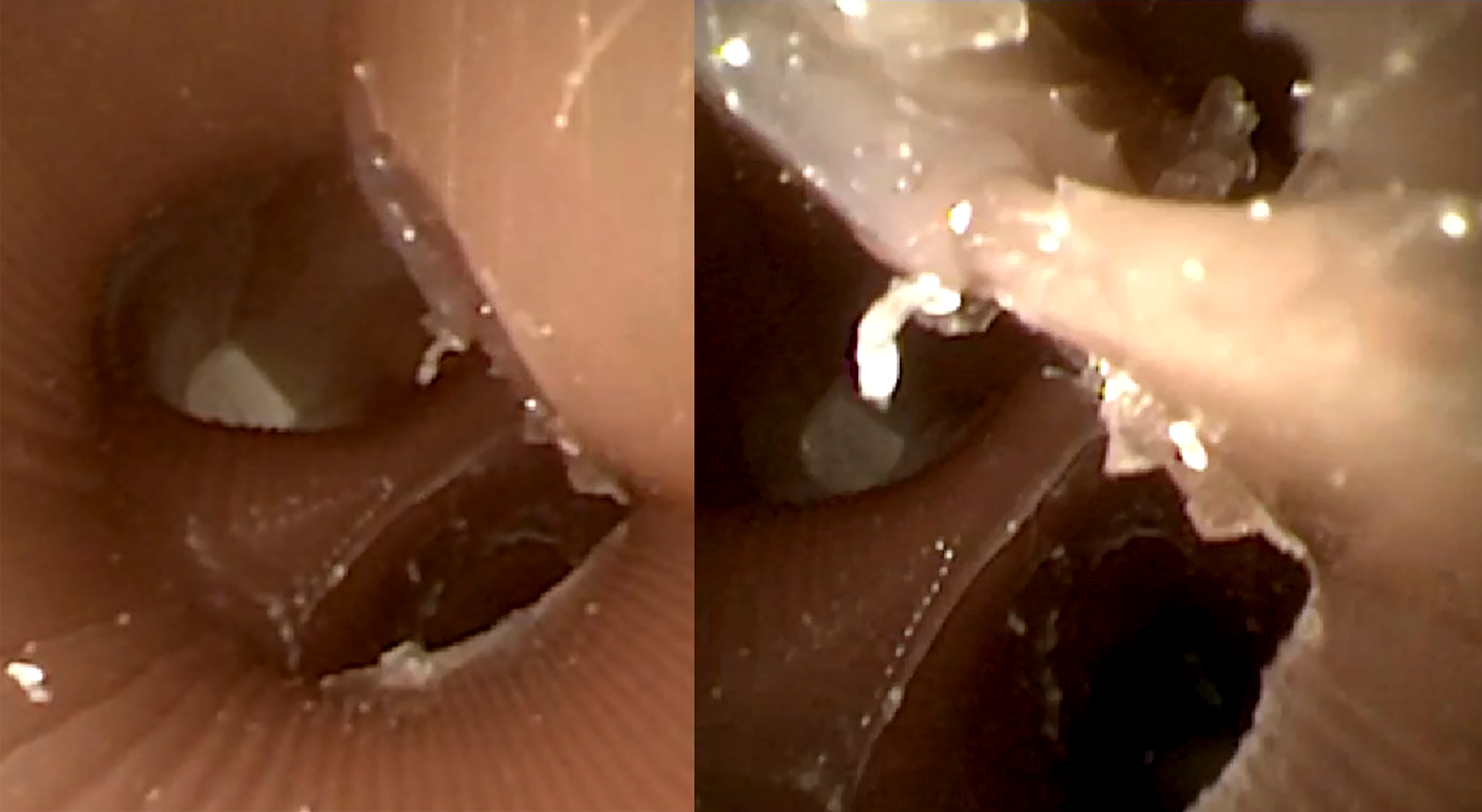

Supplement: Supplementary file 1 — Flexible bronchoscopic examination performed on the present model. Left: Visualization of the carina; Right: Right: visualization of the carina with the tracheoesophageal fistula on the upper right side of the image (TIF 5063 KB) [file 383_2024_5827_MOESM1_ESM.tif]
